# Sarcopenia as a Little-Recognized Comorbidity of Type II Diabetes Mellitus: A Review of the Diagnosis and Treatment

**DOI:** 10.3390/nu15194149

**Published:** 2023-09-26

**Authors:** Christian Salom Vendrell, Elisa García Tercero, Juan Bautista Moro Hernández, Bernardo Abel Cedeno-Veloz

**Affiliations:** 1 Hospital Universitario Doctor Peset, 46017 Valencia, Spain; 2 Hospital Universitario de la Ribera, 46600 Alzira, Spain; 3 Medical Science Liaison, Abbott Nutrición, 28050 Madrid, Spain; juan.moro@abbott.com; 4 Hospital Universitario de Navarra, 31008 Pamplona, Spain

**Keywords:** diabetes, frailty, sarcopenia, malnutrition, functional impairment, oral supplementation -HMB and exercise

## Abstract

Background: Type II diabetes mellitus (T2DM) is one of the most widespread metabolic diseases worldwide, with a significant impact on morbi-mortality. Sarcopenia has a high risk in this population (two times more risk) and a high impact at the functional level, especially in older adults. In addition, it poses enormous challenges in the diagnosis, prevention, and treatment of this disease concomitantly. The objective is to review the current knowledge on the state of muscle mass and the pathogenesis, diagnosis, and treatment of sarcopenia in people with T2DM. Methods: A bibliographic search was conducted in the PubMed-Medline databases for articles from 2015 with previously defined terms. Results: A loss of muscle mass in older diabetic patients who are malnourished or at risk of malnutrition has a proven negative impact on their autonomy and is closely related to the risk of sarcopenia as a high-impact disease, and also with frailty, as an associated multidimensional syndrome. Notably, we found that malnutrition and protein deficiency are often underdiagnosed in obese and overweight T2DM patients. Biochemical markers could help in the future with approaches to managing T2DM and sarcopenia concomitantly. The four essential elements which form the basis of care for patients with diabetes and sarcopenia are pharmacological treatment, nutrition management, regular physical exercise, and correct daily regime. Conclusions: The increasing prevalence of sarcopenia among older patients with T2DM has significant negative impacts on quality of life and is a public health concern. Effective diagnosis and management require a multidisciplinary approach involving pharmacological treatment, nutrition, exercise, and correct daily regime, with future research needed to understand the underlying mechanisms and improve diagnostic and treatment strategies.

## 1. Introduction

Diabetes mellitus is a disorder in which an alteration in the carbohydrate metabolism predominates, resulting from a decrease in pancreatic insulin secretion, a decreased peripheral sensitivity to insulin, or a variable combination of both. It is also characterized by abnormalities in the lipid and protein metabolism.

Long-term persistent hyperglycemia is not just a hallmark of T2DM, but also a major contributing factor to a wide array of organ damage. Furthermore, hyperglycemia is intricately linked with macrovascular complications, affecting larger blood vessels and leading to conditions such as coronary artery disease and stroke, as well as microvascular complications that impact smaller blood vessels, resulting in issues such as retinopathy, nephropathy, and neuropathy. The cumulative effect of these complications significantly elevates the risk of morbidity and mortality, making the management of blood sugar levels crucial for long-term health outcomes [1].

T2DM represents a major health burden for older adults, affecting approximately 25% of people over the age of 65 years worldwide, with this percentage being expected to increase further in the coming decades [1].

The VIDA study was the first to assess nutritional status in a cohort of hospitalized older patients and found a prevalence of 21.2% malnutrition and 39.1% nutritional risk [2,3]. Malnutrition worsens with age and is more pronounced in women, and is a factor that increases population-wide mortality rates. Later, the PREDyCES [4] and SeDREno [5] studies showed that diabetes is significantly associated with an increased risk of developing malnutrition.

Malnutrition may be underdiagnosed in overweight or obese patients, and dietary restrictions may even hinder nutrient intake. These restrictions often result in a negative energy/protein imbalance, increasing the risk of developing malnutrition, particularly when there is a lack of protein. Several factors contribute to the degree of malnutrition in these patients: gastroparesis due to poor intake; diabetic foot, which increases energy and protein requirements; and diabetic renal disease because of restrictions in protein intake in pre-dialysis patients [2,3]. However, the coexistence of obesity, T2DM, and sarcopenia presents a challenging health scenario, often referred to as “sarcopenic obesity”, which has severe implications for morbidity, functional decline, and quality of life [6]. The combination of obesity and a low handgrip strength suggest an increased risk of type 2 diabetes mellitus (OR 3.57, 95% CI 2.04–6.24) [7]. Understanding this triad is essential for developing comprehensive diagnostic and treatment strategies.

Older people with T2DM have higher rates of functional disability, concomitant illnesses, and common geriatric conditions, irrespective of the presence of microvascular and macrovascular complications. One of these illnesses is sarcopenia, a degenerative condition in which there is a lack of muscle or a low muscle mass, which can be a secondary to a disease state, medical condition, or to aging itself [5,8].

The prevalence of sarcopenia among individuals with T2DM is notably elevated, exhibiting a considerable range of variability. According to the existing literature, these prevalence rates fluctuate significantly, spanning from as low as 7% to as high as 29.3% [8].

**Figure 1:**
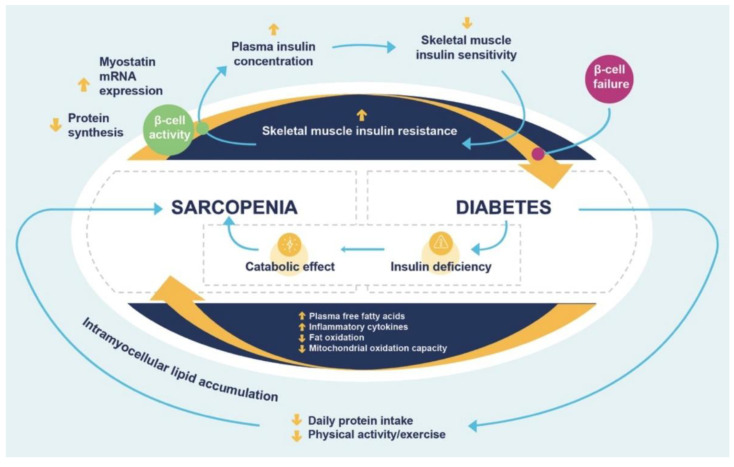
Interaction between sarcopenia and diabetes. Adapted from Landi F. et al., 2013 [9]. First, the insulin resistance of skeletal muscle is the most important link between sarcopenia and diabetes. Normal beta-cell response to insulin resistance is to enhance the secretion of insulin and the prolonged physiologic increase in the plasma insulin concentration could lead to further reduction in skeletal muscle insulin sensitivity. Second, hyperglycemia is associated with multiple metabolic abnormalities, potentially correlated with muscle cell damage, and muscle mitochondrial dysfunction leads to elevated accumulation of intramyocellular lipid metabolites. Third, insulin deficiency leads to a protein catabolic state with loss of muscle mass.

Different mechanisms may explain the higher prevalence of sarcopenia in people with T2DM. Insulin’s anabolic effect on skeletal muscle is well known; it may be gradually lost in T2DM due to the reduced insulin sensitivity associated with this disease. In addition, the decrease in insulin’s effect may lead to decreased protein synthesis and increased protein degradation, resulting in reductions in muscle mass and strength (Figure 1) [9].

In both type 1 and type 2 diabetes mellitus, muscle strength and architecture are also adversely affected, this being a pathophysiological situation that is linked to muscle mass. For all these reasons, DM is considered to be a risk factor for developing sarcopenia [10].

Chronic hyperglycemia promotes the accumulation of advanced glycation end-products (AGE) in the skeletal muscle, with a correlation between AGEs and a weakened grip strength, lower leg extension ability, and slower gait speed [9,10].

Similarly, diabetes is intricately linked with a rise in inflammatory cytokines, such as interleukin-6 and tumor necrosis factor-alpha. These elevated levels of inflammatory markers are thought to play a pivotal role in promoting the loss of muscle mass, as well as contributing to reduced muscle strength and function. The cascade of inflammation can trigger catabolic pathways in the muscle tissue, leading to protein degradation and subsequent muscle atrophy. This, in turn, can result in a diminished physical performance and functional capacity, exacerbating the already complex health challenges faced by individuals with T2DM [9,10].

T2DM is characterized by a metabolic imbalance in which ATP levels are adversely affected and mitochondria play a key role. Mitochondria are the main source of reactive oxygen species (ROS) and are essential for redox homeostasis, metabolism, and many cellular functions, including apoptosis and maintaining Ca^2+^ levels [9,10].

The relationship between mitochondria, metabolism, inflammation, and the different signaling pathways involved in this complex interaction is very important [9,10].

Alterations in the mitochondrial metabolic pathways (such as oxidative phosphorylation and the tricarboxylic acid cycle) can induce changes in gene expression that can lead to different outcomes in immune cells. For example, M1 macrophages are adversely affected in the tricarboxylic cycle and enter a pro-inflammatory state, while M2 macrophages undergo β-oxidation and produce anti-inflammatory responses [11].

**Figure 2:**
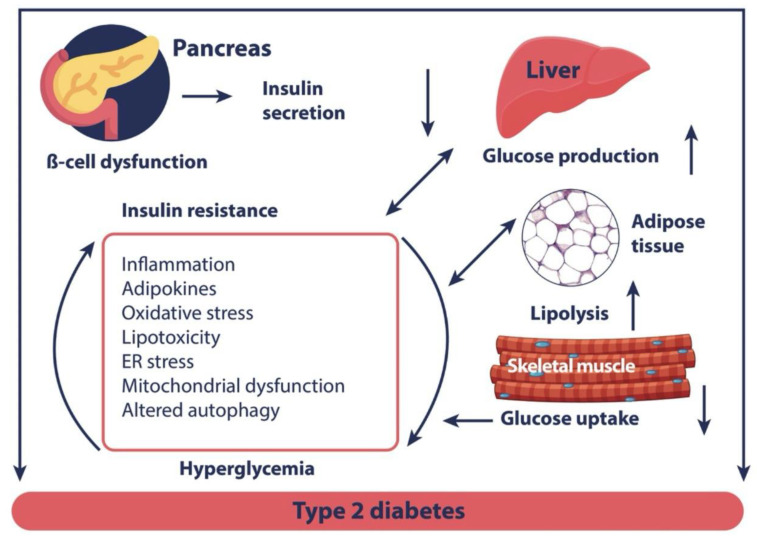
Biological mechanism potentially explaining diabetes-related muscle dysfunction [11]. Adapted from Rocha M et al., 2020 [11]. Diminished β-cell function leads to decreased insulin secretion and increased insulin resistance, affecting multiple organs and tissues. The underlying molecular mechanisms include inflammation, lipotoxicity, mitochondrial dysfunction, and endoplasmic reticulum stress.

Mitochondria play an essential role in the regulation of immune responses by modulating autophagy, endoplasmic reticulum stress, and inflammasome activation through various mechanisms, including ROS production and changes in mitochondrial DNA, which regulate and control the transcription of immune system cells. All these features underscore the importance of mitochondria in the modulation of the inflammatory response in T2DM [11] (Figure 2).

Finally, the higher prevalence of sarcopenia in T2DM is also related to the presence of macrovascular and microvascular complications, i.e., retinopathy, nephropathy, and neuropathy. Chronic kidney disease secondary to diabetic nephropathy may affect muscle mass, and diabetic peripheral neuropathy may lead to a reduction in physical activity and performance due to postural instability or vision loss. Macrovascular complications such as peripheral vascular disease can also contribute to induce muscle ischemia, as well as a poorer muscle strength, mass, and physical performance [12].

Reduced bone mass is another significant concern commonly observed in patients who fit the profile of having T2DM and sarcopenia. This decrease in bone density contributes to an elevated risk of fragility fractures. The incidence of these fractures is particularly high among women, who are already more susceptible to bone-related issues such as osteoporosis. These fractures not only pose immediate health risks, but also often lead to long-term complications, including a reduced mobility and quality of life [13,14].

The objective is to review the current knowledge on the state of muscle mass and the pathogenesis, diagnosis, and treatment of sarcopenia in people with T2DM.

## 2. Methodology

This narrative review aims to provide an in-depth analysis of the existing literature concerning the relationship between T2DM, frailty, and sarcopenia. The primary objective is to review and synthesize the information on the pathophysiology, diagnosis, and management of sarcopenia as a comorbidity in T2DM patients.

### 2.1. Literature Search

A comprehensive bibliographic search was conducted using multiple databases, including PubMed, Scopus, and Google Scholar, focusing on articles published from 2015 to present. The search terms used were “Diabetes Mellitus Type II”, “frailty”, “sarcopenia”, “pathophysiology”, “diagnosis”, and “treatment.”

### 2.2. Inclusion and Exclusion Criteria

The inclusion criteria were articles that provided insights into the relationship between T2DM and sarcopenia, its prevalence in diabetic patients, its underlying pathophysiology, and its inflammatory component. Studies that offered management recommendations or discussed the history of diabetes and diabetic control were also included. The exclusion criteria were articles that did not focus on T2DM or sarcopenia, were not peer-reviewed, or were published before 2015.

### 2.3. Review Process

After the initial search, a pool of authors reviewed the articles to assess their relevance and quality. A total of 2506 articles met the defined search criteria, and 56 were selected for the narrative review based on the selection criteria. These articles were then thoroughly reviewed to extract their key findings, which were categorized into various sub-topics such as diagnostic methods, treatment options, and future directions.

### 2.4. Data Synthesis

The data extracted from the selected articles were synthesized to provide a cohesive understanding of the relationship between T2DM, frailty, and sarcopenia. This included aspects such as the relationship between diabetes and sarcopenia, its prevalence in diabetic patients, and its pathophysiology and inflammatory components, as well as management recommendations and the history of diabetes and diabetic control.

## 3. Results

In our comprehensive literature search, we identified a total of 2506 articles that met the defined search criteria. After a rigorous review process conducted by the pool of authors, 56 articles were selected for inclusion in this narrative review. These articles were chosen based on their relevance to the key questions that we aimed to answer:

(A) What is the pathophysiology and prevalence of a loss of muscle mass and function associated with T2DM?
(B) What is the best treatment for a loss of muscle mass and function in T2DM patients?
(C) How feasible is the measurement of muscle mass and function in T2DM?

The main results are presented below:

### 3.1. Diagnostic Methods

As we have mentioned, diabetic patients are at a higher risk for sarcopenia and malnutrition. The prevalence of sarcopenia in these patients varies due to the lack of unified diagnostic criteria across the different series that have been reported (muscle strength, muscle quantity/quality, and physical performance), as well as techniques for quantitative assessments of muscle mass (dual-energy X-ray absorptiometry or bioelectrical impedance analyses) [8]; so, until a common assessment method is developed and unified, it is advisable for diagnoses to be made based on the existing available methods in each unit.

With respect to malnutrition in diabetic patients, an early nutritional assessment using any validated screening tool is essential. There are no specific tools for diabetics, although they do exist for the different settings in which they may be performed (inpatient, outpatient, or older patient), which can be seen in Table 1 [15].

The Geriatric Nutritional Risk Index (GNRI) is an indicator of nutritional status. It is a simple and accurate screening tool that includes objective factors, such as weight, height, and serum albumin. The GNRI is associated with the presence of sarcopenia in people with T2DM [16].

This index is calculated using the formula: (1.489 × albumin, g/L) + (41.7 × current weight/ideal weight).

Patients are graded as serious risk (GNRI < 82), moderate risk (GNRI 82 to <92), mild risk (GNRI 92 to 98), or no risk (GNRI > 98) [16].

It should be noted that, although many patients with T2DM are obese based on their BMI, they may also have sarcopenia, so it is important to consider more specific measures of body composition other than weight in these patients [8].

Assessments of patients’ overall functional capacity can be performed with the Senior Fitness Test battery of tests or the Short Physical Performance Battery (SPPB), which have the advantage of not requiring sophisticated equipment, being easy to use, comparison values in terms of sex and age being available and, moreover, can be applied in both the hospital and extra-hospital settings [17].

These tests provide us with a general idea of physical fitness and some of its components, but they are not very helpful for prescribing exercise loads according to results.

On the other hand, there are more reproducible and specific techniques for assessing functional performance or the physical quality that we want to work on, and these can serve as a reference for planning the workload. Among these are, for example, an estimation or measurement of the maximal oxygen uptake (peak oxygen consumption), ventilatory thresholds, actual maximum heart rate, or maximal force (one-repetition maximum, 1RM) of the muscle groups that we want to train. These techniques require more sophisticated, expensive equipment and require more physical space as well as a greater degree of specialization by the professionals who perform the assessments [17].

Key biochemical markers such as myostatin, Irisin, Brain-Derived Neurotrophic Factor (BDNF), pro-inflammatory cytokines, Growth Hormone/Insulin-like Growth Factor 1 (GH/IGF1), and testosterone play significant roles in the pathogeneses of these interconnected conditions and their pathways could, in the future, help with diagnoses [18]. For instance, Myostatin and Irisin are directly involved in muscle metabolism and can be indicative of muscle atrophy, while BDNF and pro-inflammatory cytokines are linked to inflammation, a common underlying factor in both T2DM and sarcopenia. GH/IGF1 and testosterone levels can also provide insights into the metabolic and hormonal imbalances that exacerbate these conditions. Accurate diagnoses and effective treatment strategies must, therefore, consider these biochemical markers to provide a comprehensive approach to managing T2DM and sarcopenia concomitantly.

### 3.2. Treatment

#### 3.2.1. Choosing a Glucose-Lowering Drug

**Figure 3:**
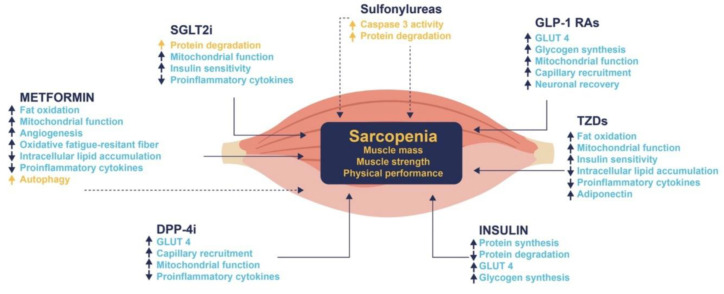
Plausible mechanisms by which glucose-lowering drugs might impact on sarcopenia acting on sarcopenia. DPP-4i, dipeptidyl peptidase-4 inhibitors; GLP-1 Ras, glucagon-like peptide-1 receptor agonists; GLUT4, glucose transporter type 4; SGLT2i, sodium-glucose transport protein 2 inhibitors; and TZDs, thiazolidinediones. ↑, increase; ↓, decrease. Blue color and continue lines indicate a beneficial effect on sarcopenia; yellow color and dotted lines indicate a detrimental effect on sarcopenia [19]. Adapted from Massimino E et al., 2021 [19].

Glucose-lowering medications appear to have a significant role in the development or mitigation of sarcopenia among patients with diabetes. These drugs vary in their impacts on muscle mass and function, largely due to differences in their mechanisms of action [19] (Figure 3):

While numerous studies have attempted to evaluate the effects of glucose-lowering medications on sarcopenia [20,21,22,23,24,25,26,27,28,29,30,31,32,33,34,35,36,37,38,39,40,41], these investigations are often marred by several limitations. These include small sample sizes, inconsistent or varying techniques for assessing sarcopenia, and a focus on sarcopenia as a secondary objective rather than as the primary focus of the study. Additionally, these studies often involve populations of varying age ranges and lack comprehensive data on comorbidities or usual physical activity levels.

Preliminary findings have suggested that insulin does not appear to have a significant effect on the development or progression of sarcopenia. Dipeptidyl peptidase-4 (DPP-4) inhibitors, on the other hand, seem to have neutral effects on muscle mass and function. Interestingly, the use of Sodium-Glucose Co-Transporter-2 (SGLT2) inhibitors has been associated with an increased likelihood of developing sarcopenia, particularly in patients already at risk [19].

Identifying patients at a higher risk for sarcopenia and choosing the most appropriate glucose-lowering drug may help reduce the risk of it developing.

#### 3.2.2. Nutritional Intervention

A mainstay of the treatment for these patients is medical nutrition therapy, both for adequate control of their diabetes and to prevent disease-related malnutrition [42].

Malnutrition is present in more than 20% of hospitalized older diabetic patients. Malnutrition in patients with diabetes worsens if not corrected and leads to a poorer prognosis and longer hospitalizations [3].

Nutrients should preferably be provided orally and, where this is not possible, via the enteral route; parenteral feeding should be reserved for those cases when the digestive route is contraindicated. As a more specialized route, post-pyloric feeding may be considered in cases where there is severe gastroparesis [43,44].

Whenever patients require artificial feeding, the enteral route, if not contraindicated, should be used, since parenteral nutrition is associated with a higher frequency of hyperglycemia and greater insulin requirements. For example, enteral nutrition should be started early for critical care patients, preferably within the first 24 h of admission, after they have achieved hemodynamic stability [43,44].

Diets specifically designed to manage hyperglycemia, which incorporate carbohydrates with a low glycemic index, high fiber content, and enrichment with monounsaturated fatty acids, have been shown to be effective in achieving optimal glycemic control. Notably, these specialized diets also have the added benefit of reducing the need for insulin, thereby offering a more holistic approach to diabetes management [45].

For patients who are grappling with both malnutrition and diabetes, it is strongly advised that their protein energy requirements be carefully calculated. This calculation should take into account various factors such as the level of physical activity engaged in by the patient and any additional disease conditions or stress factors they may be experiencing, among others. Furthermore, in scenarios where the patient is also dealing with obesity, these protein energy requirements should be adjusted to align with the patient’s weight to ensure the optimal nutritional balance and disease management [45].

With respect to the formulas used in enteral and oral nutrition, diabetes-specific formulas must have the following characteristics [46,47]: a low carbohydrate content, low glycemic index, moderate to high monounsaturated fat content in relation to the total caloric value, and high fiber content, although the effects of the latter on postprandial glycemic control do not appear to be very significant.

It should be remembered that, while there are no specific recommendations on micronutrients for patients with diabetes, supplementation with β-hydroxy-β-methylbutyrate (HMB), a metabolite of leucine catabolism, seems to be helpful in preventing muscle atrophy in diabetic patients [48].

Research has demonstrated that the implementation of outpatient nutrition counseling programs, specifically designed to prevent sarcopenia in patients with T2DM who are at a high risk, can be highly effective. These programs are not only associated with a marked decrease in the incidence of sarcopenia, but also offer a proactive approach to managing this common comorbidity in diabetic patients [46,47].

#### 3.2.3. Physical Activity

Muscle strength and architecture are affected in diabetic patients who are also at a high risk for developing myopathy. It is therefore important and necessary to begin an exercise training program based on therapeutic exercises from the earliest stages of their diabetes in order to achieve better glycemic control, prevent the progression of other complications, and increase the active life expectancy of these patients [49].

The American Diabetes Association guidelines [50] recommend physical exercise as part of diabetes treatment, as it helps to improve glycemic control, as well as improve the control of cardiovascular risk factors, weight, and a better quality of life.

Traditionally, different types of exercise are classified in binary terms as endurance or strength exercises. However, this classification is oversimplified. Further classifications of exercise are metabolically related (aerobic vs. anaerobic) or related to the type of muscle contraction: isotonic, when the contraction against a force causes the length of the muscle to shorten (concentric) or lengthen (eccentric), and isometric (static or without change in muscle length).

Aerobic exercise is a form of physical activity that involves sustained and repetitive movements engaging the large muscle groups, primarily aimed at improving cardiovascular and respiratory function. This category includes a variety of activities, such as walking, cycling, jogging, and swimming, each offering different levels of intensity and specific health benefits. Resistance or strength training, on the other hand, focuses on building muscle mass and enhancing muscle strength. This type of training incorporates a range of exercises that utilize free weights, weight machines, body weight, or resistance bands to target specific muscle groups. Flexibility exercises are designed to improve the range of motion in the joints, thereby enhancing overall mobility. These exercises are particularly beneficial for maintaining joint health and preventing stiffness. Balance exercises aim to improve stability and coordination, which are crucial for gait and fall prevention. These exercises are especially important for older adults or individuals with conditions that affect balance. Activities such as tai chi and yoga offer a holistic approach by combining elements of flexibility, balance, and resistance training. These activities not only improve physical health, but also have the added benefit of enhancing mental well-being [50] (Table 2).

In hospitalized older adults with DM, multicomponent exercise interventions, together with the Vivifrail exercise program [50], have been found to be effective in improving performance status, as measured with the SPPB, in addition to improving handgrip strength and functionality.

Regular cardiorespiratory exercise increases insulin sensitivity, reduces time of hyperglycemia, and results in a reduction in glycosylated hemoglobin (HbA1c) by between 0.3% and 0.6% [51]. Performed at a high intensity, these cardiorespiratory exercises have greater benefits for glycemic control than if they are performed at a low intensity [52].

On the other hand, resistance training increases lean muscle mass, which, in turn, is known to be related to insulin sensitivity. Hence, this form of exercise is of greater importance to diabetic patients with pre-sarcopenia [53].

For individuals with T2DM to lose visceral fat, a moderately high volume of exercise (approximately 500 kcals) performed 4–5 times per week is necessary [54].

#### 3.2.4. Correct Daily Regime

The endogenous circadian system modulates the timing of behavioral rhythms and physiological processes. There are interindividual differences in the timing of circadian rhythms defined as chronotypes and these are related to health and behavioral problems, especially when evening types are compared to morning types [55] Evening types, when compared to morning types, are significantly associated with diabetes (OR 1.73; 95% CI, 1.01–2.95), metabolic syndrome (OR 1.74; 95% CI, 1.05–2.87), and sarcopenia (OR 3.16; 95% CI, 1.36–7.33), especially in men.

Among the interventions for correcting daily regime, nutrition, physical activity, and behavioral therapy stand out as the key components of lifestyle interventions for resetting chronotypes [56], improving glycemic control, and preventing or treating T2DM and sarcopenia.

Within the dietary recommendations, which are known as chrononutrition, are a Mediterranean-style dietary pattern, macronutrient composition with a low glycemic index and load carbohydrates, high in fiber and protein and accompanied by fat and/or protein to reduce glucose and insulin spikes, and a food sequence of first consuming low-energy dense foods that contain water, such as soups, vegetables, or fruits, followed by protein-rich foods and then starch-rich foods to improve glycemic and insulin responses [56].

In relation to exercise, because the time of day when exercise is performed can influence its effect on circadian rhythm, mitochondrial function, and muscle performance, a personalized exercise prescription is recommended according to the chronotype of each individual [57].

Behavioral therapy uses cognitive and behavioral techniques to help people change their eating habits, physical activity, and stress management. It provides regular feedback and support from a trained professional, either in person or through technology, to improve the adherence to and maintenance of a healthy life plan. Improving the adherence to interventions for diabetes and sarcopenia is key to their effectiveness [56].

## 4. Discussion

Diabetes is a major health issue due to its high incidence and prevalence, as well as the impact of increased morbidity and mortality on patients afflicted with it.

A loss of muscle mass in older diabetic patients who are malnourished or at risk for malnutrition has a demonstrably negative impact on their autonomy. It is closely related to the risk of sarcopenia, a high-impact disease, and also to frailty, a multidimensional syndrome associated with adverse health events and, ultimately, disability [58,59].

The prevalence of malnutrition is high in both institutionalized and hospitalized older adults. However, preventive measures can be instituted when older patients live in their own homes, so it is worth choosing tools that allow for assessments to be made of the risk for malnutrition presented by each patient. This should be conducted not only at the time of screening, but also on a continuous basis once the nutrition intervention begins. The first difficulty that arises is the validation of the questionnaire, considering the heterogeneity of the population and the lack of a single model or standard test [58,59]. 

From a clinical point of view, it is extremely important to implement improvements in the methods used to identify sarcopenia and its associated risk factors in patients with T2DM, and hence to institute therapeutic strategies focused on adequate intake and regular physical activity and correct daily regimes in a timely manner [60].

In light of the current review, it is crucial to adopt a multidimensional approach to the screening, prevention, and management of T2DM and sarcopenia. Screening methods should ideally involve medical history, physical exams, and nutritional assessments. The Short Physical Performance Battery (SPPB) stands out as a practical approach to sarcopenia for assessing functional capacity due to its simplicity and easy reproducibility. Regarding pharmacological interventions, the potential modification of hypoglycemic drugs should be evaluated on a case-by-case basis, with SGLT2 inhibitors being the only ones directly related to sarcopenia. Dietary interventions can play a significant role, with high-protein diets and HMB showing promise in improving muscle mass. Exercise regimens should be tailored to individuals, focusing on multicomponent exercise interventions. Vivifrail offers an exercise program based on SPPB that improves the functional capacity in this population. Special attention should be given to the timing of insulin administration to avoid hypoglycemia during physical activity. Behavioral therapy employs cognitive and behavioral techniques to help people to change their eating habits, physical activity levels, and stress management strategies. These considerations form the basis of a comprehensive approach to managing these complex conditions.

Due to the scant evidence available, trials aimed at developing specific physical activity programs and interventions using supplementation for patients with this profile are needed.

## 5. Future Directions

The current review sheds light on the under-recognized issue of sarcopenia as a comorbidity in patients with T2DM. While we explored the existing literature to understand the pathophysiology, prevalence, diagnostic methods, and treatment options, there are several avenues for future research that could provide more comprehensive insights into this complex relationship.

### 5.1. Pathophysiology

Understanding the molecular mechanisms that link T2DM and sarcopenia could be a fertile ground for future research. Identifying specific biomarkers and pathways could lead to targeted therapies that can mitigate the effects of sarcopenia in T2DM patients.

### 5.2. Diagnostic Methods

The review highlighted various diagnostic methods such as dual-energy X-ray absorptiometry and bioelectrical impedance analyses. However, there is a need for more cost-effective and accessible diagnostic tools that can be used in primary care settings.

### 5.3. Treatment Options

While we discussed the roles of glucose-lowering medications and nutritional interventions, future studies should focus on the efficacy of combining these treatments with physical exercise and other lifestyle modifications such as correct daily regimes. Clinical trials comparing different treatment modalities could provide valuable data.

### 5.4. Prevalence

The wide range of prevalence rates (7% to 29.3%) indicates a need for large-scale epidemiological studies to obtain a more accurate picture. Understanding the factors contributing to this variability can help with targeted interventions.

### 5.5. Technology Integration

The use of wearable technology for the continuous monitoring of muscle mass and function could offer real-time data, aiding in early diagnoses and treatment adjustments.

### 5.6. Patient-Centered Approaches

Future research should also focus on the quality of life and functional outcomes from the patient’s perspective. This could involve developing patient-reported outcome measures specific to T2DM and sarcopenia.

By addressing these areas, we can hope to build a more comprehensive understanding of sarcopenia in the context of T2DM, ultimately leading to better diagnostic and treatment strategies that can improve patient outcomes.

## 6. Conclusions

The prevalence of sarcopenia is increasing among older patients with T2DM. Different mechanisms may explain the association between the two, such as impaired insulin sensitivity, chronic hyperglycemia, the accumulation of advanced glycation end-products (AGEs), subclinical inflammation, and microvascular and macrovascular complications.

The high negative impact of this situation on quality of life, affecting physical and psychosocial health, makes it a public health concern.

The four essential elements that form the basis of care for patients with diabetes are pharmacological treatment, nutrition management, regular physical exercise, and correct daily regime.

Health professionals should be vigilant about sarcopenia, paying special attention to older and/or diabetic patients, in order to implement the appropriate nutritional, pharmacological, and physical activity measures. With this, we will be able to reduce the prevalence, severity, and disability of this geriatric syndrome in the older population. Future research should focus on elucidating the molecular mechanisms linking T2DM and sarcopenia, developing cost-effective diagnostic tools, and evaluating the efficacy of combined treatment modalities. These efforts aim to provide a comprehensive understanding that can improve diagnostic accuracy and patient outcomes.

## Figures and Tables

**Table 1:** Parameters required for the diagnosis of undernutrition/malnutrition.

**Diagnostic Method**	**Objective**
Medical history	Assess history for malabsorption: previous GI procedures, taste disorders, anorexia, nausea, vomiting, gastrointestinal motility disorders, dysphagia, oral health issues, history of pulmonary aspiration, diabetic gastroparesis, allergies, drug use, nutritional supplements, and medications, etc.
Physical examination	Assess loss of fat and muscle in specific body regions: orbital, temporal, and intercostal spaces, etc Muehrcke’s lines in the fingernails suggest hypoalbuminemia, alopecia is associated with protein deficiency, and scaling of the scalp results from essential fatty acid deficiency.
Anthropometric data	Calculate body mass index (BMI) and percentage of unintentional weight loss.
Nutrition assessment	Use questionnaires to assess nutritional risk: Nutritional Risk Screening 2002 (NRS 2002), Subjective Global Assessment (SGA), Mini Nutritional Assessment (MNA), Malnutrition Universal Screening Tool (MUST), and Short Nutritional Assessment Questionnaire (SNAQ).
Bioelectrical methods	Bioelectrical impedance analysis (BIA) provides a detailed description of body composition (fat mass, fat-free mass, body water, lean body mass, and vector analysis).
Imaging tests	The use of muscle ultrasonography to assess the quadriceps provides a simple, quick, and cost-effective way of estimating total body muscle quantity and quality, but the cut-off points for this tool have not yet been validated in the population.
Biochemical laboratory markers	Changes in biochemical parameters such as ghrelin, leptin, adiponectin, myostatin, cancer-associated fibroblast (CAF), tumor necrosis factor-alpha (TNF-a), Interleukin-1 (IL-1), Interleukin-6 (IL-6), growth hormone (GH)/Insulin-like growth factor I (IGF-1), and testosterone.

**Table 2:** Quality classification of physical activities.

**Aerobic activities**	• Walking
	• Swimming
	• Dancing
	• Cycling
	• Ellipticals
	• Low-impact aerobics
	• Water aerobics
**Strength exercises**	• Resistance band exercises
	• Self-loading or with a load
	• Climbing stairs
	• Sit-to-stand
	• Carrying things
	• Some tai chi exercises
	• Yoga
**To improve** **balance or** **neuromotor** **fitness**	• Balance
	• Agility
	• Coordination
	• Gait
	• Proprioceptive training
	• Multifaceted activities: tai chi and yoga
**Flexibility**	Take joints through a set of range of motion exercises at the start of each session and perform different sets of stretches at various times throughout the session

## Data Availability

Not applicable.
